# Rosai-Dorfman Disease: Rare Pulmonary Involvement Mimicking Pulmonary Langerhans Cell Histiocytosis and Review of the Literature

**DOI:** 10.1155/2018/2952084

**Published:** 2018-04-05

**Authors:** Rashid AL Umairi, Danielle Blunt, Wedad Hana, Matthew Cheung, Anastasia Oikonomou

**Affiliations:** ^1^Department of Medical Imaging, Sunnybrook Health Sciences Centre, University of Toronto, Toronto, ON, Canada; ^2^Department of Medicine, Sunnybrook Health Sciences Centre, University of Toronto, Toronto, ON, Canada; ^3^Department of Anatomic Pathology, Sunnybrook Health Sciences Centre, University of Toronto, ON, Canada

## Abstract

Rosai-Dorfman disease (RDD), also known as sinus histiocytosis with massive lymphadenopathy, is a rare nonmalignant lymphohistiocytic proliferative disorder. We report a patient with RDD who presented with multiple skin lesions, pulmonary involvement, and CT manifestations mimicking Langerhans cell histiocytosis, which improved after initiation of corticosteroid treatment.

## 1. Introduction

Rosai-Dorfman disease (RDD), also known as sinus histiocytosis with massive lymphadenopathy, is a rare nonmalignant lymphohistiocytic proliferative disorder, described for the first time by Rosai and Dorfman in 1969 [1]. The disease typically manifests as painless, massive lymphadenopathy that frequently affects cervical lymph nodes and is accompanied by fever, leukocytosis, increased erythrocyte sedimentation rate, and hypergammaglobulinemia [2]. Although the disease commonly affects lymph nodes, extranodal involvement, either isolated or associated with lymph node involvement, has been reported in up to 43% of cases including skin, soft tissue, nasal cavity, bone, orbit, intrathoracic structures, central nervous system, breast, urogenital tract, and gastrointestinal tract [3–13]. We report a case of RDD with multiple skin lesions and pulmonary CT manifestations mimicking Langerhans cell histiocytosis, which improved after initiation of corticosteroid treatment.

## 2. Case Report

**Figure 1:**
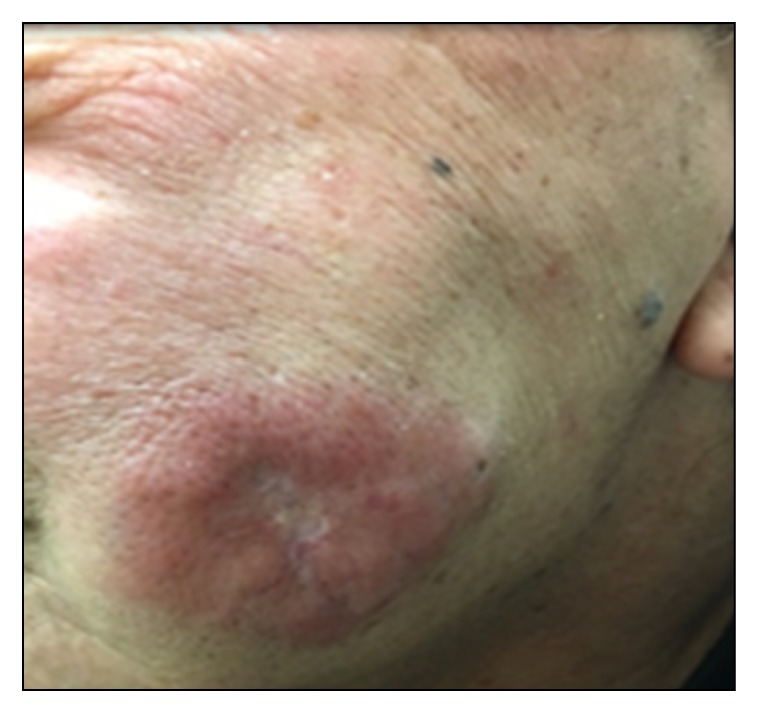
Heterogeneous pink plaque localized to the left cheek.

**Figure 2:**
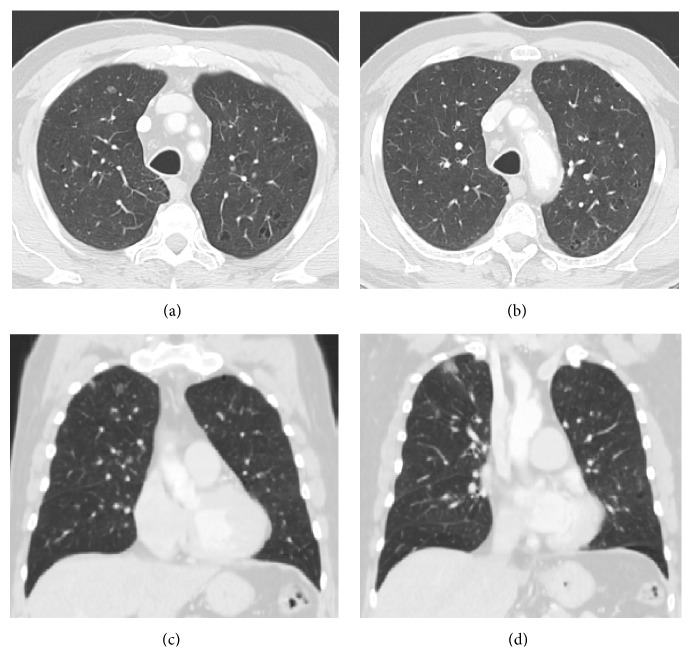
Selected axial and coronal HRCT images on lung windows show bilateral pulmonary cysts. Some of them are rounded and some demonstrate bizarre shapes with multiloculation and internal septations. There are also bilateral ground-glass nodules ranging from 1-2 mm up to a few centimeters. The findings are upper lobe predominant and spare the lung bases.

**Figure 3:**
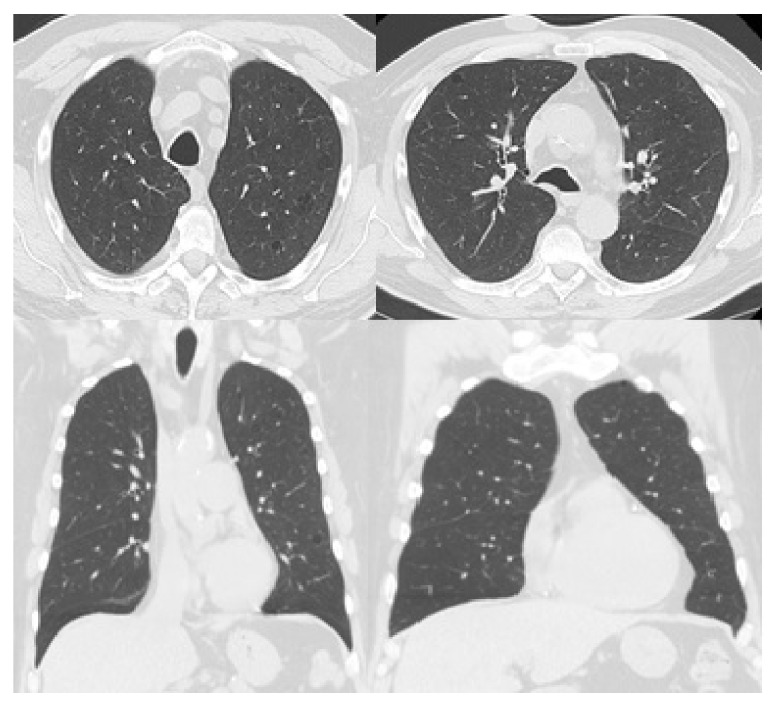
Follow-up HRCT images at the same level and planes as Figure 2 show resolution of the ground-glass nodules with persistent lung cysts.

A 65-year-old male, smoker with a history of type 2 diabetes mellitus, hypertension, and hyperlipidemia, started to notice slowly growing lesions in his left cheek, posterior left shoulder, and left upper arm. The lesions were not tender or itchy. There was no history of shortness of breath. On physical examination, an indurated 10 × 10 cm lesion arising on the left cheek was identified (Figure 1). Similar lesions were also identified in the right upper arm measuring 2 × 3 cm and posterior left shoulder measuring 5 × 5 cm. No palpable lymphadenopathy or organomegaly was noted. Subsequent biopsy and microscopic examination of the left cheek skin lesion revealed an infiltrate formed by sheets of foamy macrophages surrounded by a dense plasma cell infiltrate and lymphocytes with scattered neutrophils. There was focal granuloma formation. Special stains for organism were negative. Immunohistochemical studies showed diffuse staining of the histiocytes for S100 protein and CD68. They were negative of CD1a, ruling out histiocytosis X. The lymphocytes were a mixture of T and B cells. The plasma cell infiltrate was polyclonal and was confirmed by in situ hybridization for kappa and lambda. The combination of findings was consistent with cutaneous Rosai-Dorfman disease. CT scan of the chest was performed, which showed bilateral thin and mildly thick-walled pulmonary cysts. Some of them were rounded and some demonstrated bizarre shapes with multiloculation and internal septations. There were also numerous ground-glass nodules ranging from 1-2 mm up to a few centimeters. The findings had a characteristic upper lung zone predominance sparing the lung bases and the anteromedial parts of the middle lobe and lingula (Figure 2). There was no intrathoracic lymphadenopathy and no pleural effusion. Initially, the skin lesion at the cheek was treated with radiotherapy (1500 cGy in 5 fractions); however, there was only minimal response. The patient was started on 50 mg oral prednisone daily, which resulted in improvement of the skin lesions. However, treatment with prednisone was complicated by worsening of diabetes and peripheral edema and hence was stopped. A follow-up chest CT five months later showed complete resolution of the ground-glass nodules and persistence of the lung cysts (Figure 3).

## 3. Discussion

Rosai-Dorfman disease (RDD) is a rare nonmalignant histiocytic/phagocytic cell proliferative disorder, which typically presents in childhood and early adulthood with male predominance and a higher incidence in African-Americans [2, 6]. The histiocytic disorders are classified based on the cell origin. For instance, RDD and Langerhans cell histiocytosis (LCH) are both abnormal proliferation of histiocytic/phagocytic cells; however, they are different entities. RDD is monocytic/macrophage proliferative disorder, whereas LCH is dendritic cell proliferation disorder [14, 15].

The etiology of Rosai-Dorfman disease remains unknown and different etiologies have been proposed including viral infection and immune dysfunction. A proposed mechanism is cytokine activation following HHV6 or EBV infection leading to activation and accumulation of histiocytes, which is supported by the expression of HHV-6 antigens in abnormal histiocytes from RDD patients [2, 6, 8, 16].

Patients with Rosai-Dorfman disease typically present with fever and massive painless cervical lymphadenopathy. Extranodal involvement, either isolated or associated with lymph node involvement, has been reported in up to 43% of cases [2, 17]. Our patient had no abnormally enlarged intrathoracic or intra-abdominal lymphadenopathy. Approximately 10% of the patients have skin lesions, with 3% having disease only detectable in the skin. Skin lesions usually are xanthoma-like, yellowish, or reddish-brown plaques or nodules that can ulcerate. Our patient presented with slowly growing skin lesions on his left cheek, posterior left upper shoulder, and left upper arm (Figure 1). Intrathoracic lymphadenopathy is the most common intrathoracic manifestation in RDD, reported in 66% of patients in one case series (21). In that study, mediastinal adenopathy was the most common location of intrathoracic lymphadenopathy seen in 5 out of 21 patients. Less common findings included pleural effusion and interstitial infiltration with peripheral and basal predominance, while honeycombing and cystic changes were minimal or absent [18]. Intrathoracic manifestations of Rosai-Dorfman disease have rarely been reported in the literature, in less than 3% of cases, characterized by pulmonary nodules, pleural effusion, cystic changes, and interstitial lung disease. However, no details are available about the interstitial findings [19–21]. Ji et al. reported a case with Rosai-Dorfman disease presenting with left lower lobe mass [22]. Park et al. reported a case of Rosai-Dorfman disease with mediastinal and hilar lymph nodes and bilateral irregular nodular and patchy ground-glass opacities [23]. CT chest of our patient showed bilateral pulmonary cysts and numerous ground glass nodules of variable size ranging from a few millimeters to a few centimeters with characteristic upper lobe predominance and sparing of the lung bases and anteromedial parts of the middle lobe and lingula. There was no intrathoracic lymphadenopathy or pleural effusion. The chest CT findings were identical to the expected radiological features for pulmonary Langerhans cell histiocytosis [20]. However, the biopsy of the concomitant skin lesion confirmed the histological diagnosis of RDD. There was no histologic biopsy of the pulmonary lesions; however it would be less likely, although not excluded, that the pulmonary lesions would be histologically consistent with Langerhans cell histiocytosis when the skin lesion showed Rosai-Dorfman disease.

The diagnosis of RDD is based on a combination of clinical findings and characteristic histological features. The two classical histological features include scattered histiocytes with emperipolesis, which represent histiocytic phagocytosis of intact plasma cells, lymphocytes, and cellular debris as well as expression of S100 protein, CD14, CD68, and CD1c and absence of staining for CD1a and MHC-2 (which are both positive for Langerhans-type dendritic cells). The differential diagnosis includes Langerhans cell histiocytosis, lymphoma, immunoglobulin G4-related interstitial lung disease, and Erdheim-Chester disease. Our patient had a biopsy of his skin lesion, which showed focal granulomas formation with histiocytes positive for S100 protein and CD68 and negative for CD1a, confirming RDD and excluding pulmonary Langerhans cell histiocytosis. Cartin-Ceba and his group reported two patients with lung parenchymal findings and lung biopsy-proven Rosai-Dorfman disease. The first patient had bilateral intestinal infiltrates and the second patient had bilateral subpleural reticulation with scattered areas of ground glass opacities and tiny cysts. Histopathologic findings revealed interstitial thickening due to abnormal histiocytic collections and associated mixed inflammatory infiltrates [18].

RDD is a self-limiting disease; however, fatality has been rarely reported due to massive adenopathy, involvement of different organs, anemia, and immunological abnormalities [24]. Approximately 50% of the patients require no treatment and spontaneous remission has been reported in approximately 20% of the cases [14, 25, 26]. Treatment is preserved for symptomatic patients or patients with vital organ or system involvement. If patients require treatment, surgery is an appropriate option for localized disease with complete remission reported in RDD cases involving the CNS treated with surgery only [27, 28]. Steroids, chemotherapy, and radiation therapy have been used for systemic disease or in cases of extensive organ involvement with varying degree of success [2, 27, 28]. Our patient had local radiotherapy to the skin lesion with no significant response. Subsequent oral prednisone was associated with improvement of the skin lesion and resolution of the ground-glass nodules.

## 4. Conclusion

We present a case with a skin lesion histologically confirmed as Rosai-Dorfman disease (RDD) and pulmonary CT pattern identical to pulmonary Langerhanss cell histiocytosis. Familiarity with this rare pulmonary CT manifestation of RDD along with the appropriate clinical findings may raise suspicion and accurate diagnosis of this rare type of histiocytosis.
